# What are survivorship care plans failing to tell men after prostate cancer treatment?

**DOI:** 10.1002/pros.24116

**Published:** 2021-03-23

**Authors:** Youngjee Choi, Katherine C. Smith, Aishwarya Shukla, Amanda L. Blackford, Phuoc T. Tran, Kimberly S. Peairs, Thomas M. DeMarco, Amanda Choflet, Kristen Farling, Madeline Kelso, Michael A. Carducci, Nancy Mayonado, Claire F. Snyder

**Affiliations:** 1 Johns Hopkins University School of Medicine, Baltimore, Maryland, USA; 2 Johns Hopkins Bloomberg School of Public Health, Baltimore, Maryland, USA; 3 Johns Hopkins Sidney Kimmel Comprehensive Cancer Center, Baltimore, Maryland, USA; 4 University of Washington School of Medicine, Seattle, Washington, USA; 5 Tidal Health Richard A. Henson Research Institute, Salisbury, Maryland, USA; 6 San Diego State University, San Diego, California, USA; 7 Johns Hopkins Medical Institution, Baltimore, Maryland, USA

**Keywords:** cancer guidelines, cancer survivorship, prostate cancer, survivorship care plans

## Abstract

**Background:**

Survivorship care plans contain important information for patients and primary care physicians regarding appropriate care for cancer survivors after treatment. We describe the completeness of prostate cancer survivorship care plans and evaluate the concordance of follow-up recommendations with guidelines.

**Methods:**

We analyzed 119 prostate cancer survivorship care plans from one academic and one community cancer center, abstracting demographics, cancer/treatment details, and follow-up recommendations. Follow-up recommendations were compared with the American Cancer Society (ACS), American Society of Clinical Oncology (ASCO), and National Comprehensive Cancer Network (NCCN) guidelines.

**Results:**

Content in >90% of plans included cancer TNM stage; prostate-specific antigen (PSA) at diagnosis; radiation treatment details (98% of men received radiation); and PSA monitoring recommendations. Potential treatment-specific side effects were listed for 82% of men who had surgery, 86% who received androgen deprivation therapy (ADT), and 97% who underwent radiation. The presence of posttreatment symptoms was noted in 71% of plans. Regarding surveillance follow-up, all guidelines recommend an annual digital rectal exam (DRE). No plans specified DRE. However, all 71 plans at the community site recommended at least annual follow-up visits with urology, radiation oncology, and primary care. Only 2/48 plans at the academic site specified follow-up visits. All guidelines recommend PSA testing every 6-12 months for 5 years, then annually. For the first 5 years, 90% of plans were guideline-concordant, 8% suggested oversurveillance, and 2% were incomplete. In men receiving ADT, ACS and ASCO recommend bone density imaging and NCCN recommends testosterone levels. Of 77 men on ADT, 1% were recommended bone density imaging and 16% testosterone level testing.

**Conclusions:**

While care plan content is more complete for demographic and treatment summary information, both sites had gaps in reporting posttreatment symptoms and ADT-related testing recommendations. These findings highlight the need to improve the quality of information in care plans, which are important in communicating appropriate follow-up recommendations to patients and primary care physicians.

## 1 | INTRODUCTION

For the estimated 3.6 million prostate cancer survivors in the United States, survivorship care plans (SCPs) are one approach to improving care provision after completing cancer treatment.^1^ SCPs are an important tool for communicating essential cancer-related information from oncology to the patient and primary care provider (PCP).^2^ While SCPs vary in their format, they typically cover two categories of information: (1) about one’s cancer and subsequent treatment and (2) follow-up recommendations for future care. To simplify and streamline SCPs and promote their completion, the American Society of Clinical Oncology (ASCO) developed a clinical expert statement that identified key SCP components for summarizing treatment-related details and providing a plan for follow-up visits and testing.^3^

Separate from recommendations provided in SCPs, societies have also published cancer-specific survivorship guidelines defining appropriate clinical follow-up for cancer survivors. For prostate cancer, these guidelines have been put forth by the American Cancer Society (ACS), ASCO, and National Comprehensive Cancer Network (NCCN).^4–6^ Actual follow-up recommendations within SCPs, influenced by providers’ clinical judgment, may vary from cancer-specific guidelines.

Because the quality of information and actual recommendations included in SCPs have the potential to impact their effectiveness, we analyzed both aspects of SCPs in prostate cancer patients enrolled in a larger study comparing modalities of SCP delivery. We (1) describe the completeness of SCP content and (2) evaluate the guideline concordance of SCP follow-up recommendations.

## 2 | MATERIALS AND METHODS

### 2.1 | Study design

We conducted a content analysis of SCPs from a randomized controlled trial of SCP delivery. While the primary analysis will evaluate receipt of care recommended in SCPs, this analysis describes the completeness of prostate cancer SCPs and evaluates whether SCP recommendations are concordant with ACS, ASCO, and NCCN guidelines.

### 2.2 | Study sites

Two sites recruited trial participants: (1) an urban, academic, tertiary medical center with separate clinics based on the cancer type and specialty and (2) a rural, regional community medical center with one general oncology program. Before this trial, neither site was systematically delivering SCPs to prostate cancer survivors.

### 2.3 | Study participants

Study participants were survivors of newly diagnosed or recurrent Stage I–III prostate cancer, with the following eligibility criteria: age ≥21 years; cancer treated with intent to cure; ability to complete study data collection in English; and medical insurance. Patients were recruited around treatment completion. All patients were recruited from the radiation oncology practices at the two sites because the structure of the urology practices made it difficult to identify eligible patients.

### 2.4 | SCP templates and completion

Prostate cancer SCP templates in this study were based on the ASCO prostate cancer template,^7^ with each study site making its own adaptations and creating an electronic version in the electronic medical record (EMR) Epic^™^. The templates had the following general structure: (1) patient contact and demographic information; (2) healthcare provider information including cancer team members; (3) a treatment summary, including cancer diagnosis information; completed treatments including surgery, radiation, chemotherapy, and androgen deprivation therapy (ADT); ongoing ADT; persistent treatment-related symptoms; and (4) a follow-up care plan, including a schedule of clinical visits, cancer-related testing, possible issues for survivors, and health promotion recommendations. Sections 2–4 included key components recommended by the ASCO clinical expert statement.^3^

SCPs were completed by a member of the oncology team: six nurses at the academic site and three nurses at the community site. While the study team made ASCO and other relevant follow-up guidelines available, clinicians had full discretion in completing the SCPs, including recommended follow-up.

### 2.5 | Data abstraction

We used the ASCO template and ASCO clinical expert statement on care planning to develop a data abstraction form for capturing SCP components, which were then entered into a REDCap database.^8^ Data abstracted from a random 10% sample of plans were reviewed by a second individual, with <1% error rate in the original abstraction. The second reviewer corrected any identified errors.

### 2.6 | Analysis

We used descriptive statistics to summarize patient and SCP characteristics. Study sites were compared to identify differences in patterns of care between the academic (urban) and community (rural) practice using Fisher’s exact test, Wilcoxon rank-sum tests, and t tests. Care plan and participant characteristics were also compared between SCPs with prostate-specific antigen (PSA) oversurveillance recommendations and SCPs that were guideline-concordant. SCP recommendations for physical exam and PSA/bone density/testosterone testing were qualitatively compared with the 2014 ACS,^4^ 2015 ASCO,^5^ and 2019 NCCN^6^ guidelines; as the NCCN revises its guidelines at least annually, recommendations across 2017, 2018, and 2019^6,9,10^ were compared and found to have no substantive changes in follow-up surveillance recommendations. SCP recommendations were categorized as undersurveillance (less frequent follow-up than advised by guidelines), concordant with guidelines, or oversurveillance (more frequent follow-up than advised by guidelines). *p* < .05 was considered statistically significant. Analyses were completed using R version 3.6.0.^11^

## 3 | RESULTS

### 3.1 | Study population

A total of 142 prostate cancer patients enrolled in the study; 119 SCPs were analyzed (48 from the academic site, 71 from the community site). Of the 23 patients excluded from the analysis, 3 withdrew consent, 8 became ineligible, and 12 were determined to be ineligible post-enrollment. All SCPs were completed within 6 months of treatment completion, between January 2017 and February 2019.

Patients had a median age of 69 years (Table 1). Approximately half of the men had T1 stage disease. Notably, 98% of patients received radiation treatment (2% had incomplete documentation for radiation treatment); 18% of men had surgery, and 65% received ADT (77 men completed and/or had ongoing ADT).

### 3.2 | SCP content compared with the ASCO template

#### 3.2.1 | General content from the ASCO template across all SCPs

Based on the ASCO template (verbatim or with minimal modification), a number of fields were present across all SCPs. Patients were recommended to see their PCP for age-appropriate general health care, including cancer screening tests. After the follow-up care plan, a standard section listed 13 specific issues a prostate cancer survivor may experience, with a recommendation to contact providers for additional assistance. At the end of the SCP, cancer.net was listed as a resource; 59% of plans listed additional resources.

#### 3.2.2 | General content beyond the ASCO template across all SCPs

The following additions were made to the ASCO template built into Epic^™^, with the same language used across all SCPs. Whereas the ASCO template has a “yes/no” with free text option to list persistent treatment-related symptoms, the Epic^™^ template had a dropdown menu of nine categories of side effects (urinary incontinence, urinary urgency/frequency/hesitancy, rectal urgency/frequency, erectile dysfunction, fatigue, hot flashes/flushes, decreased sex drive, moodiness, weight gain), with an additional “other” option with free text. There was a general advisement to report any new or different symptoms lasting beyond 2 weeks to one’s provider. While the institution and contact information for care providers were not included, a phone number to contact if the patient was “not sure who to contact” was provided across all plans; the community site also included contact information for their institution’s medical and radiation oncology clinics.

#### 3.2.3 | Patient-specific content

Several patient-specific key components were consistently provided (>90% of the time) across both sites (Table 1): age, relevant providers (PCP, radiation oncologist); diagnosis date; TNM stage; PSA at diagnosis; and radiation details (type, end date, irradiated location). For other key SCP components, documentation varied. For example, cancer-specific details were provided in different ways across the two sites. While both sites provided TNM staging and PSA at diagnosis at similarly high rates, the community site reported prognostic staging preferentially while the academic site reported the Gleason score more often.

Documentation of potential and actual treatment-related side effects also varied (Table 1). In the section outlining potential late and long-term treatment effects, possible radiation side effects were provided in 97% of SCPs in which men received radiation. Possible surgical and ADT-related effects were listed at lower rates (82% and 86%, respectively).

A separate SCP section was dedicated to documenting persistent treatment-related side effects experienced by patients. This section on posttreatment side effects was completed in 87% of plans, and completed more frequently at the community site (94% compared with 75% at the academic site). However, nearly a quarter of community site SCPs reported that patients had no persistent symptoms (whereas relatively fewer [6%] reported an absence of symptoms at the academic site). Of the total 119 plans, at least one persistent side effect was documented in 84 SCPs (71%). The rate of documenting at least one persistent side effect was similar across the two sites (69% at the academic site, 72% at the community site).

The documented persistent symptoms from treatment are outlined in Table 2. Across all SCPs, 39% of men were noted to experience erectile dysfunction, 38% urinary symptoms, and 15% bowel-related symptoms. Other symptoms listed as possible late and long-term effects in the original ASCO prostate cancer SCP template,^7^ particularly related to ADT, were not recorded for any men: anemia, bone thinning, breast growth or tenderness, cognitive changes, increased cholesterol, and loss of muscle mass. At the academic site, 29/33 SCPs that documented any persistent side effects provided additional comments regarding the symptoms: 27 outlined a timeframe in which symptoms would be expected to continue and improve, 4 advised a timeframe in which persisting symptoms required follow-up, and 1 provided medication recommendations. None of the community site SCPs included similar comments.

Health promotion recommendations were provided 85% of the time (Table 3). In SCPs where health promotion was included, both sites consistently (>90%) provided general recommendations regarding diet and nutrition, alcohol use, physical activity, and sunscreen use. The community site provided recommendations for smoking cessation and weight management significantly more often than the academic site; cardiovascular health was addressed solely at the community site (76%). Advice around medication management was only provided at the academic site (though only in 15% of SCPs addressing health promotion). Notably, both sites did not provide health promotion recommendations in a number of other domains including vaccinations and mental health.

### 3.3 | Concordance of follow-up recommendations with clinical guidelines

#### 3.3.1 | Follow-up visits and exam

All guidelines recommend an annual digital rectal exam. No plans specified this exam, but all 71 plans at the community site recommended at least annual follow-up visits with urology, radiation oncology, and primary care. Part of the community site recommendation specified a visit to the “radiation oncologist/urologist every 6 months for 3 years then annually for 5 years.” However, this recommendation is ambiguous, as it is unclear if the stated follow-up frequency/duration was with each of the providers (the intended interpretation based on conversations with clinicians) or if seeing either of the two providers at the specified frequency was being recommended. The academic site specified follow-up visits in only 2/48 SCPs, both of which recommended visits with radiation oncology every 6 months. The academic site provided an Epic^™^-generated list of future appointments that was imported as part of the “schedule for clinical visits” (88% at that site), but this list did not provide a schedule of frequency/duration for follow-up visits.

#### 3.3.2 | PSA testing

All guidelines recommend PSA testing every 6–12 months (optional every 3 months if high risk per NCCN) for 5 years, then annually. PSA testing recommendations were provided in 98% of plans (46/48 at the academic site, 71/71 at the community site). The duration was not specified in any SCPs. If applied to the first 5 years, 90% of SCP recommendations were guideline-concordant (3% included every 3 months × 2 followed by every 6 months), 8% suggested oversurveillance (nine plans at the academic site included a recommendation to check 1 month after treatment), and 2% did not provide any recommendations. SCPs with PSA oversurveillance recommendations did not differ according to TNM stage, Gleason score, or age compared with those with guideline-concordant PSA recommendations (*p* > .05 for all comparisons).

#### 3.3.3 | Follow-up for men on ADT

ACS and ASCO guidelines recommend both baseline imaging and fracture risk assessment tool (FRAX) score calculation, while the NCCN guideline recommends baseline bone density imaging based on a FRAX score. For checking testosterone levels, ACS and ASCO guidelines do not specify any recommendations while the NCCN guideline recommends achieving castration levels without specifying a frequency in testing.

Of 77 men who completed or had ongoing ADT, 1 individual was recommended for bone density imaging, no one had FRAX scores documented, and 12 men (16%) had a recommendation for testosterone levels.

## 4 | DISCUSSION

In our analysis of prostate cancer SCPs, we aimed, first, to describe the completeness of SCP content in comparison to the ASCO template and ASCO SCP expert statement and, second, to evaluate the concordance of SCP follow-up recommendations with clinical guidelines. Our analysis suggests that while SCP documentation was relatively consistent for demographic and treatment summary information, gaps exist in follow-up recommendations, which are critical for primary care physicians to be aware of to provide appropriate, individualized care for cancer survivors.

Several patterns were notable in SCP follow-up recommendations, likely related to site-specific use of template defaults. Whereas follow-up visit recommendations at the community site were the same across all SCPs, follow-up schedules were largely incomplete at the academic site. Additionally, while PSA recommendations were largely guideline-concordant, all of the oversurveillance recommendations were from the academic site. Lastly, there were gaps in documenting symptoms at treatment completion, recommending non-PSA follow-up testing, and providing health promotion recommendations.

Providers completed templates that were site-specific, which led to certain patterns of documentation. The community site prepopulated a default visit schedule and PSA recommendations. While community site providers had the option to modify these defaults, they did not, such that the recommendations were uniform across all SCPs. These defaults prevented the lack of follow-up visit recommendations seen in the majority of plans at the academic site, where all surveillance recommendations were free text. At the academic site, providers primarily drove recommendation patterns, which may have led to PSA recommendations that constitute oversurveillance. While it is important to use clinical judgment in tailoring care to the individual patient, it is notable that variation in PSA recommendations across SCPs was not accounted for by individual patient characteristics. It is also possible that providers at the academic center disagreed with guidelines, but there was no apparent pattern to when their recommendations deviated from guidelines. The differences between the two sites suggest that template defaults may help prevent both undersurveillance (e.g., follow-up visit) and oversurveillance (PSA) recommendations.

Documented treatment-related side effects may also be related to how templates were built. For symptoms included in a dropdown menu on the SCP template (e.g., erectile dysfunction, urinary symptoms, and bowel symptoms), reported rates were within ranges cited in the literature, which although vary widely. Our SCPs showed that 39% of men reported erectile dysfunction, 38% reported urinary symptoms, and 15% reported bowel symptoms. In comparison, men who received radiation in the Prostate Testing for Cancer and Treatment trial (most similar to our patients of whom 98% received radiation), 18% experienced erectile dysfunction, 65% nocturia, and 3.5% bloody stools at 6 months posttreatment.^12^ Similarly, in a large survey of prostate cancer survivors, among men who received radiation, 54%–63% reported erectile dysfunction, 3%–20% reported urinary symptoms, and 0%–11% reported bowel symptoms at 6 months posttreatment.^13^ Meanwhile, in our study, other symptoms, particularly related to ADT, were not included in the dropdown menu of the SCP template, and were not recorded for any men. Men who received ADT also did not consistently get a list of potential treatment-related effects. In contrast, potential effects of radiation were reported at a rate of 97%; this may have been influenced by the fact that SCPs were completed exclusively by radiation oncology nurses.

Gaps in follow-up recommendations related to ADT led to care recommendations constituting undersurveillance. These recommendations may also benefit from defaults written into templates (e.g., “baseline DEXA scan if ADT received” for bone density testing). Because of the numerous variables needed to calculate a FRAX score, an EMR decision tree may be the most effective approach to calculate it and identify those at higher fracture risk. Because guidelines vary on their recommendations for assessing bone density and testosterone, having better alignment across guidelines may also help reconcile recommendations in SCPs.

A section on health promotion was missing in 15% of SCPs, more often at the academic site. Smoking cessation, weight management, and cardiovascular health were less consistently addressed at the academic site; certain domains were not included in any SCPs. While the reason for these variations in health promotion categories is unclear, having template defaults may not only help ensure that health promotion is captured in the SCP but also increase the consistency and breadth of recommendations incorporated.

Of note, there appeared to be one benefit of not using template defaults. At the academic site, 88% of SCPs that listed posttreatment symptoms provided additional documentation of expectations and management strategies (compared with none at the community site). This finding suggests that we need to balance using template defaults in some sections of the SCP (e.g., follow-up recommendations that align with clinical guidelines) while providing individualized, patient-specific information in other areas.

Our study had several limitations. The study was conducted only at two sites, and all of our patients were recruited from radiation oncology. Our patients were less representative of all prostate cancer survivors, in whom radiation is used in 27% of low-risk cancer patients.^14^ However the differences in SCPs from the two sites demonstrate the potential for using template defaults to provide more complete and consistent documentation across multiple providers. While the Commission on Cancer is no longer requiring SCP completion by institutions, SCPs remain one of the main tools to coordinate care and communicate follow-up recommendations after cancer treatment.^15^ Therefore, having a method to improve the reliability of SCP content and guideline concordance across providers will continue to be important. It may be argued that cancer specialists are providing guideline-concordant, high-quality care for their patients that was not captured in the documentation of our SCPs due to well-documented barriers to care plan completion.^3^ However, as PCPs take on greater roles in the care of cancer survivors and as patients desire knowledge and control of their care, these care plans serve an important role in providing essential information about follow-up care after cancer treatment.

## 5 | CONCLUSIONS

SCP content is more complete for demographic and treatment summary information, with gaps in addressing treatment effects, follow-up recommendations, and health promotion. In particular, follow-up for men receiving ADT could be enhanced by better addressing posttreatment symptoms, potential ADT effects, and bone density assessment. While the community site provided guideline-concordant recommendations for PSA testing, there were patterns of oversurveillance at the academic site, suggesting a role for template defaults in providing uniform recommendations. Clear and consistent SCP documentation is needed to guide and educate patients and PCPs in addressing necessary care after cancer treatment.

## Figures and Tables

**TABLE 1:** Care plan and patient characteristics

	All patients *N* = 119	Academic site *N* = 48	Community site *N* = 71	*p* value
Patient date of birth specified, *n* (%)	119 (100)	48 (100)	71 (100)	–

Patient median age (range)	69 (46, 84)	68 (49, 84)	69 (46, 80)	.49

Date of diagnosis specified, *n* (%)	119 (100)	48 (100)	71 (100)	–

TNM stage specified, *n* (%)^a^	108 (91)	42 (88)	66 (93)	.35
T1	57 (53)	22 (52)	35 (53)	.16
T2	29 (27)	8 (19)	21 (32)	
T3	21 (19)	11 (26)	10 (15)	
T4	1 (1)	1 (2)	0 (0)	

Prognostic stage specified, *n* (%)	73 (61)	5 (10)	68 (96)	<.01

NCCN risk category specified, *n* (%)	9 (8)	9 (19)	0 (0)	<.01

Gleason score specified, *n* (%)	54 (45)	45 (94)	9 (13)	<.01

Grade group specified, *n* (%)	15 (13)	2 (4)	13 (18)	.03

Gleason or grade group specified, *n* (%)	67 (56)	45 (94)	22 (31)	<.01

PSA at diagnosis specified, *n* (%)	116 (98)	47 (98)	69 (97)	>.99

Primary care provider specified, *n* (%)	117 (98)	48 (100)	69 (97)	.51

Received radiation, *n* (%)^b^	116 (98)	47 (98)	69 (97)	>.99
Radiation oncologist specified	116 (100)	47 (100)	69 (100)	–
Radiation type specified	105 (90)	37 (79)	68 (99)	<.01
Radiation end date specified	114 (98)	45 (96)	69 (100)	.16
Radiation location specified	112 (97)	43 (92)	69 (100)	.02

Had surgery, *n* (%)^c^	22 (18)	10 (21)	12 (17)	.39
Urologist specified	20 (91)	8 (80)	12 (100)	.20
Date of surgery specified	20 (91)	10 (100)	10 (83)	.48

Completed ADT, *n* (%)^d,e^	37 (31)	29 (60)	8 (11)	<.01
Urologist specified	16 (43)	8 (28)	8 (100)	<.01
Radiation oncologist specified	37 (100)	29 (100)	8 (100)	–
Medical oncologist specified	1 (3)	1 (3)	0 (0)	>.99
Specialist other than cancer team specified^f^	1 (3)	1 (3)	0 (0)	>.99
ADT end date specified	31 (84)	27 (93)	4 (50)	.01
ADT agent specified	36 (97)	28 (97)	8 (100)	>.99

Ongoing ADT, *n* (%)^d,e^	56 (47)	19 (40)	37 (52)	.26
Urologist specified	41 (73)	5 (26)	36 (97)	<.01
Radiation oncologist specified	56 (100)	19 (100)	37 (100)	–
Medical oncologist specified	4 (7)	3 (16)	1 (3)	.11
ADT end date specified	32 (57)	19 (100)	13 (35)	<.01
ADT agent specified	55 (98)	19 (100)	36 (97)	>.99

Potential treatment-related side effects specified, *n* (%)	118 (99)	47 (98)	71 (100)	>.99
Yes, not by treatment type	5 (4)	5 (11)	0 (0)	
Yes, by treatment type^g^	113 (96)	42 (89)	71 (100)	
Radiation	108 (93)	39 (83)	69 (100)	<.01
Surgery	18 (82)	6 (60)	12 (100)	.03
ADT^e^	66 (86)	24 (73)	42 (95)	<.01

Actual treatment-related side effects specified,^h^ *n* (%)	103 (87)	36 (75)	67 (94)	<.01
Section not completed	16 (13)	12 (25)	4 (6)	<.01
0 side effects reported	19 (16)	3 (6)	16 (22)	
≥1 side effect reported	84 (71)	33 (69)	51 (72)	
Comments or management strategy listed for side effect(s)	29 (34)	29 (88)	0 (0)	<.01

EMR imported future appointments listed, *n* (%)	42 (35)	42 (88)	0 (0)	<.01

Schedule of follow-up visits specified, *n* (%)	73 (61)	2 (4)	71 (100)	<.01

Abbreviations: ACS, American Cancer Society; ADT, androgen deprivation therapy; ASCO, American Society of Clinical Oncology; EMR, electronic medical record; NCCN, National Comprehensive Cancer Network; PSA, prostate-specific antigen.

a Denominators for the rows below are those who had TNM stage specified.

b Denominators for the rows below are those who received radiation.

c Denominators for the rows below are those who received surgery.

d Denominators for the rows below are those who received ADT.

e 16 men who completed some portion of their ADT were also receiving ongoing ADT at the time of SCP completion; a total of 77 men ever received ADT.

f Only one specialist other than the cancer team (geriatrician) was specified across all SCPs.

g Denominators for the rows below are those who received radiation, surgery, and ADT (completed and/or ongoing) respectively.

h Denominators for the row s below are the total SCPs for the two sites.

**TABLE 2:** Actual persistent treatment-related side effects

	All patients	Academic site	Community site	*p* value
Fatigue,^a^ *n* (%)	54 (45)	31 (65)	23 (32)	<.01
Erectile dysfunction,^a^ *n* (%)	46 (39)	22 (46)	24 (34)	.25
Mood changes,^a^ *n* (%)	41 (34)	22 (46)	19 (27)	.05
Reduced sexual desire,^a^ *n* (%)	24 (20)	0 (0)	24 (34)	<.01
Weight changes,^a^ *n* (%)	18 (23)	0 (0)	18 (41)	<.01
Urinary symptoms,^b^ *n* (%)	44 (38)	11 (23)	33 (48)	.01
Rectal symptoms,^b^ *n* (%)	17 (15)	8 (17)	9 (13)	.60
Leg swelling,^b^ *n* (%)	0 (0)	0 (0)	0 (0)	–
Hot flashes,^c^ *n* (%)	25 (32)	2 (6)	23 (52)	<.01
Anemia,^c^ *n* (%)	0 (0)	0 (0)	0 (0)	–
Bone thinning,^c^ *n* (%)	0 (0)	0 (0)	0 (0)	–
Breast growth/tenderness,^c^ *n* (%)	0 (0)	0 (0)	0 (0)	–
Cognitive changes,^c^ *n* (%)	0 (0)	0 (0)	0 (0)	–
Increased cholesterol,^c^ *n* (%)	0 (0)	0 (0)	0 (0)	–
Loss of muscle mass,^c^ *n* (%)	0 (0)	0 (0)	0 (0)	–

Abbreviation: ADT, androgen deprivation therapy.

a Patients who received surgery, radiation, or ADT are included (*N* = 119 all patients, *N* = 48 academic site, *N* = 71 community site).

b Patients who underwent surgery or radiation are included (*N* = 116 all patients, *N* = 47 academic site, *N* = 69 community site).

c Patients who received ADT are included (*N* = 77 all patients, *N* = 33 academic site 1, *N* =44 community site).

**TABLE 3:** Health promotion recommendations (101/119 [85%] of care plans contained these recommendations)

	All patients *N* = 101	Academic site *N* = 34	Community site *N* = 67	*p* value
Diet and nutrition, *n* (%)	101 (100)	34 (100)	67 (100)	–
Alcohol use, *n* (%)	100 (99)	34 (100)	66 (99)	>.99
Physical activity and exercise, *n* (%)	98 (97)	34 (100)	64 (96)	.55
Sunscreen use, *n* (%)	96 (95)	33 (97)	63 (94)	.66
Smoking cessation, *n* (%)	91 (90)	28 (82)	63 (94)	.08
Weight management, *n* (%)	88 (87)	25 (74)	63 (94)	<.01
Cardiovascular health, *n* (%)	51 (50)	0 (0)	51 (76)	<.01
Management of medications, *n* (%)	5 (5)	5 (15)	0 (0)	<.01
Bone health, *n* (%)	0 (0)	0 (0)	0 (0)	–
Sleep, *n* (%)	0 (0)	0 (0)	0 (0)	–
Body image, *n* (%)	0 (0)	0 (0)	0 (0)	–
Mental health, *n* (%)	0 (0)	0 (0)	0 (0)	–
Sexual health, *n* (%)	0 (0)	0 (0)	0 (0)	–
Vaccinations, *n* (%)	0 (0)	0 (0)	0 (0)	–
Management of other illnesses, *n* (%)	0 (0)	0 (0)	0 (0)	–

## Data Availability

Data will be made available upon request.
